# Autoinflammatory Diseases and Cytokine Storms—Imbalances of Innate and Adaptative Immunity

**DOI:** 10.3390/ijms222011241

**Published:** 2021-10-18

**Authors:** Annalisa Marcuzzi, Elisabetta Melloni, Giorgio Zauli, Arianna Romani, Paola Secchiero, Natalia Maximova, Erika Rimondi

**Affiliations:** 1 Department of Translational Medicine, University of Ferrara, 44121 Ferrara, Italy; annalisa.marcuzzi@unife.it (A.M.); giorgio.zauli@unife.it (G.Z.); arianna.romani@unife.it (A.R.); 2 LTTA Centre, Department of Translational Medicine, University of Ferrara, 44121 Ferrara, Italy; elisabetta.melloni@unife.it (E.M.); erika.rimondi@unife.it (E.R.); 3 Bone Marrow Transplant Unit, Institute for Maternal and Child Health-IRCCS Burlo Garofolo, 34137 Trieste, Italy; natalia.maximova@burlo.trieste.it

**Keywords:** autoinflammatory disease, cytokines, hyperinflammation, NLRP3, inflammation

## Abstract

Innate and adaptive immune responses have a well-known link and represent the distinctive origins of several diseases, many of which may be the consequence of the loss of balance between these two responses. Indeed, autoinflammation and autoimmunity represent the two extremes of a continuous spectrum of pathologic conditions with numerous overlaps in different pathologies. A common characteristic of these dysregulations is represented by hyperinflammation, which is an exaggerated response of the immune system, especially involving white blood cells, macrophages, and inflammasome activation with the hyperproduction of cytokines in response to various triggering stimuli. Moreover, hyperinflammation is of great interest, as it is one of the main manifestations of COVID-19 infection, and the cytokine storm and its most important components are the targets of the pharmacological treatments used to combat COVID-19 damage. In this context, the purpose of our review is to provide a focus on the pathogenesis of autoinflammation and, in particular, of hyperinflammation in order to generate insights for the identification of new therapeutic targets and strategies.

## 1. Introduction

Autoinflammation, as an autoimmune response, is due to the excessive activation of the immune system and shows a clinical phenotype characterized by alternating periods of exacerbation and remission [1]. It is known that innate and adaptive immune responses are closely associated, and related pathologies are the direct consequence of an incorrect balance between these two responses [2].

The main differences in the deregulation of these two compartments of the immune system are the components and the relative cells involved. As widely known, the pathogenesis of autoimmunity is characterized by a defect in adaptive immunity and the consequent production of autoantibodies with the participation of T and B lymphocytes, while, conversely, autoinflammation involves innate immunity [3,4,5,6] and presents inflammatory episodes in the absence of both autoreactive T cells and high autoantibody titer [7].

In particular, the cells of the innate immune system (epithelial and dendritic cells, polymorphonuclear leukocytes, and macrophages) play a double role in the environment of autoimmune diseases: they act not only as an immediate barrier to the inflammatory process but also as effectors in the evolution of the inflammatory response.

The condition of hyperactivity of innate immunity usually manifests itself on a monogenic basis, and it is caused by the mutations of genes that codify for different proteins involved in the regulatory mechanisms of the inflammatory response: these are named “monogenic” or “hereditary” autoinflammatory diseases [8,9]. This mechanism could also be applied to pathological conditions identified as “multifactorial autoinflammatory syndromes” [10]. This double effect is possible because innate immunity cells recognize molecular patterns associated with both pathogens and tissue damage. The different mutations of pathogenic pathways, in fact, induce real molecular and receptor recruitment in order to determine a condition of hyperactivity of innate immunity.

In 2007, McGonagle et al. established that immunological disorders should be conceived as a “*continuum*” between monogenic autoinflammatory forms and monogenic autoimmune forms. To date, there is clear evidence that autoinflammatory diseases can have both an adaptive and an autoimmune component. At the base of the monogenic forms, there would be an activation of the adaptive immunity system: an example is represented by the differentiation of CD4+ cells in Th17 in cryopyrin-associated autoinflammatory syndromes (CAPS), which decreases in response to IL-1 blockage [11,12].

The pleiotropic role of T cell effectors (Th1 and Th17) in the mechanism of autoinflammation is also evident in the pathogenesis of atherosclerosis [13]: the T-mediated immune response in atherosclerosis presents an altered balance between effector T cells and Treg cells, and the effects of these cells lead to vulnerable plaques that can break and cause thrombotic events [14,15]. Both cell types play a central role in inflammation associated with atherosclerotic lesions. The alteration of the balance between Treg and Th cells could reflect on their phenotypic plasticity, determining a differentiation between regulatory and inflammatory phenotypes [16,17]. In addition to the plasticity of Treg, in the autoinflammatory mechanism, it should not be underestimated that T effectors in many pathological conditions can differentiate toward more pathogenic phenotypes during the progression of disease. For example, under the influence of innate inflammatory cytokines, protective Th17 cells lose the ability to produce IL-10 and become capable of producing IL-22 and IFNγ by acquiring a pathogenic phenotype [18,19]. This transformation of Th17 cells into pathogenic effector cells is promoted by cytokines expressed in the atherosclerotic plate (IL-1β, IL-6, and IL-23) and, thus, promotes hyperinflammation [20,21].

The aim of this review is therefore to describe the main elements on which the pathogenesis of autoinflammatory diseases is based in order to try to provide evidence demonstrating that they may be useful in the diagnostic and therapeutic approach of pathological conditions with the same clinical epiphenomenon. More specifically, the focus of this manuscript is to describe how the inflammasome platform, hyperinflammation, and cytokine secretion may be useful for identifying effective therapeutic targets for other diseases with the same involvement.

## 2. Autoinflammatory Disease: Since the Beginning of ’90s a New Branch of Medicine

Autoinflammatory diseases are a branch of medicine that was officially born in 1997, when the Mediterranean fever gene family was first identified (OMIM# 249100) [22].

Already in 1949, Siegal S. first described the symptoms of this disease concerning a patient with a peritonitis attack associated with unusual symptoms [23], and, as such, the historic term for familial Mediterranean fever was Siegal-Cattan-Mamou syndrome [24]. Hobart Reiman subsequently wrote a more complete description of this disease, using the definition “periodic disease” for the first time [25].

At the end of 1990, besides Mediterranean familial fever, two other diseases with recurrent or periodic fevers were added to the auto-inflammatory diseases: hyper-IgD (hyperimmunoglobulin syndrome D, HIDS) and TRAPS (periodic syndrome associated with tumor necrosis factor receptor 1) [26,27,28]. Over the past 20 years, an increasing number of auto-inflammatory diseases, with great heterogeneity and generally an early onset, have been discovered [29,30,31,32].

Autoinflammatory pathologies are characterized by a chronic course, and numerous sufferers are burdened from damages in the long term. Some pharmacological treatments for these pathologies have been available for a few years, and these drugs are able to control the main symptoms and allow subjects affected by these genetic diseases to have an acceptable quality of life, although for the most severe symptoms, there are still no effective treatments. In most cases, the pharmacological treatments used are new biological drugs that act by blocking the molecules of inflammation produced in excess in the acute phase of inflammatory epiphenomenon [33].

These diseases present a clinical picture that varies naturally according to the specific causative genetic mutations, and they are characterized by the presence of continuous or sub-continuous inflammation caused by the release of specific cytokines, with onset in the first months/years of life presenting with fever, joint involvement, skin manifestations, and serological markers [34]. In particular, the symptom that these autoinflammatory pathologies have in common is precisely the fever that suddenly presents itself in full health, with almost always a very high body temperature (39–40 °C).

The inflammation that characterizes these diseases represents a form of defense of the organism from pathogens, and it is strongly associated with mutations of a protein complex referred to as inflammasome. The recognition of foreign molecules determines the transduction of the intracellular signal, which induces the expression of genes, including interferon *(IFN)-α, IFN-β, TNF (Tumor Necrosis Factor)*, gene sequences of interleukin 1 (IL-1), and other cytokines that represent disease markers whose identification is crucial, and could represent specific therapeutic targets.

### 2.1. Role of the Inflammasome in the Pathogenesis of Autoinflammatory Diseases

In 2002, for the first time, Jürg Tschopp et al. identified the inflammasome as an intracellular multiprotein complex that assembles in response to molecular patterns associated with pathogens (PAMPs—pathogen-associated molecular patterns) and cellular or tissue damage of various nature (DAMPs—danger-associated molecular patterns) that is able to induce an inflammatory reaction [35].

To date, we know that the processes activated by inflammasomes are of great importance not only in an antimicrobial response but also in the regular metabolic pathways and immune reactions [36]. Inflammasomes, in fact, are formed in the cytosolic compartment of immune and inflammatory cells as an immune response to exogenous and endogenous signals [37].

These complexes originate in the presence of a disorder condition caused by biological, physical, chemical, and metabolic agents (e.g., deposition of uric acid crystals in gout, cholesterol in arteriosclerosis, free fatty acids and lipids in obesity, and beta-amyloid protein in Alzheimer’s disease) and high reactive oxygen species (ROS) levels; moreover, they are involved in the reduction of the cytosolic concentration of potassium ions and other factors. Inflammasomes are able to integrate a multitude of signals and converge them into pro-inflammatory responses.

In the last 20 years, several inflammasomes have been identified [38]; however, the currently most characterized one is the NLRP3 (NOD-like receptor protein 3) intracellular inflammasome, which belongs to the NOD-like receptor (NLR) family. It is activated in response to several signals and plays an important role in inflammatory diseases, autoimmune diseases, metabolic diseases (metabolic syndrome, obesity, diabetes, and gout), cardiovascular diseases, neurodegenerative diseases (Parkinson’s and Alzheimer’s disease), multiple sclerosis, and also in psychiatric diseases [39,40,41,42].

Recently, great attention has been given to the study of natural and synthetic compounds that are able to modulate NLRP3 inflammasome in different inflammatory diseases. Among these molecules, gallic and butyric acids, for example, demonstrated their potential in modulating NLRP3 markers in intestinal inflammation [43], while the JAK (Janus kinase) inhibitor tofacitinib ameliorates symptoms in rheumatoid arthritis by inhibiting NLRP3 inflammasome [44].

NLRP3 belongs to the pattern recognition receptor (PRR) family of innate immunity that can detect signals from intracellular antigens and that are expressed in numerous immune cells (tissue macrophages, dendritic cells, epithelial cells, neutrophils, and adaptive immunity cells) [45]. Toll-like receptors (TLRs) belong to the PRR group and are expressed on the surface of the cell membrane and can be activated by molecules associated with pathogens (PAMPs) and harmful signals in the presence of inflammatory non-bacterial damages (DAMPs). Both types of molecules are able to overcome the cell membrane and also directly stimulate the cytoplasmic cell receptors of the innate immune system. These sensors include the NLRs that have a PYD domain (pyrin domain), a caspase activator, and a CARD domain (caspase activation and recruitment domain). NLRP3, in particular, is expressed in the cytosol of innate immunity cells (circulating monocytes, tissue macrophages, dendritic cells, and neutrophils) and inflammatory cells.

This multiprotein complex induces the activation of inflammatory caspase-1 that, in turn, activates interleukin-1β (IL-1β) and interleukin-18 (IL-18) cytokines, which give rise to a systemic inflammatory response. In addition, the activation of caspase-1 can induce a form of inflammatory cell death called pyroptosis.

Unlike most inflammatory cytokines that are regulated by selective transcription and expressed as mature proteins, IL-1β and IL-18 are produced as pro-proteins and require cleavage on inflammasome before their release and activity.

IL-1β is a multifunctional cytokine capable of acting on almost all types of cells and organ systems in the human body. The secretion and concentration of circulating IL-1β and IL-18 play a pleiotropic role in the immune response. First of all, they represent a response to infection, but, at the same time, an excess of their concentration can lead to great toxicity, manifesting itself as a systemic inflammatory response syndrome, and to an increase in mortality [46,47].

The activation of inflammasome NLRP3 can be attributed to multiple causes, such as the deregulation of mitochondrial activity resulting in the production of ROS, which is also associated with a decrease in cytoplasmic potassium levels [48,49,50]. In addition, the transcription factor NF-kB (Nuclear Factor kappa-light-chain-enhancer of activated B cells) also has a regulatory role in the activation of inflammasomes and can contribute to the initiation and development of inflammatory diseases, such as rheumatoid arthritis, inflammatory bowel disease (IBD), and multiple sclerosis [51,52].

Finally, several studies suggested an important role of ubiquitylation in NLRP3 inflammasome regulation. Transcription/transduction inhibition is, indeed, not effective in preventing inflammasome triggering and activation; otherwise, NLRP3 ubiquitination critically regulates its stability and function, and, as a consequence of a stimulus, NLRP3 ubiquitination increases. It is however necessary to underline that these data are in contrast with the evidence that NLRP3 undergoes de-ubiquitylation in response to pro-inflammatory signals, inducing overall post-transcriptional changes that activate NLRP3 [37].

### 2.2. Cytokines in the Auto-Inflammatory Diseases

Cytokines are a broad group of proteins and glycoproteins that are produced and secreted by different cell types, especially by lymphocytes and macrophages, in response to growth, differentiation, and cell death stimuli [53,54].

Cytokines include different growth factors involved in immune cell proliferation, and, based on their functional activity, we can distinguish interferons and TNF family members as regulators of innate and acquired immunity, chemokines involved in the recruitment and chemotaxis of leukocytes during inflammation, and, lastly, interleukins that act on leukocytes. Moreover, cytokines can be distinguished as primary and secondary cytokines. Primary cytokines are synthesized immediately after the antigenic stimulus, while secondary cytokines are produced following the stimulation of the primary ones [55].

In the contest of autoinflammatory diseases, cytokines play a crucial role because they are responsible for the onset of systemic inflammation in the absence of infectious triggers [56].

The hyperreactivity of innate immunity, which characterizes these pathologies, is in most cases secondary to the mutations of genes encoding for proteins crucial in the regulation of inflammatory responses supported by inflammasome activation [57]. The cytokine that characterizes the typical inflammation of these autoinflammatory pathologies is IL-1β, a primary pro-inflammatory cytokine, which plays a fundamental role both in innate immunity and inflammatory responses [58,59]. This cytokine is secreted by different cell types, among which we can mention monocytes/macrophages, dendritic cells, fibroblasts, and endothelial cells. Its synthesis as an inactive precursor (pro-IL1β) is caused by bacterial infections, by the presence of TNF, or by the interaction of cells that secrete it with CD4+ T cells [60]. The active form of IL-1β is obtained with proteolytic cleavage by caspase-1. This protease is in turn activated by the stimulation of NLR proteins and the consequent assembly of the inflammasome. Moreover, recent studies have shown that the release of IL-1β is mediated by the receptor for extracellular ATP, P2X7, capable of controlling potassium efflux from cells [61,62,63]. Other studies also suggest that the P2X7 receptor is implicated in the process of maturation from pro-IL1β to IL-1β through the activation of caspase-1 [64].

The important role of this cytokine comes from the fact that it is involved in several biological processes as a regulator of immunity and inflammatory responses, inducing the expression of cyclooxygenase-2 (COX-2) and acute phase proteins, such as C-reactive protein (PCR), serum amyloid-A (SAA), fibrinogen, and several protease inhibitors.

Another cytokine structurally homologous to IL-1 is IL-18, whose receptor belongs to the superfamily of the IL-1R toll-like receptors but it is functionally very different from IL-1 [65]. IL-18 has multiple biological effects: it induces the production of IFN-γ in NK cells (antitumor activity), B cells, CD8, and macrophages, and it is a powerful inductor of NO, showing marked antimicrobial activity toward intracellular pathogens [66]. Since IL-18 can induce the production of chemokines of CC and CXC types and of IL-1β and TNF-α, it plays a particularly effective role in inflammation.

Similar to IL-1β, it is synthesized as an inactive precursor and then activated by caspase-1 or similar caspase-1 enzymes (activated by Fas/Fas-l) induced by inflammasome. IL-18, in synergy with IL-12, induces the synthesis of IFN-γ in NK cells as a response against infections by intracellular microorganisms, but, at the same time, hyperproduction of IL-12 and IL-18 can induce severe phlogistic alterations, and, as such, IL-18 can be considered a proinflammatory cytokine [67]. This cytokine seems to play a key role in many inflammatory conditions: there are a lot of data in the literature that support a strategic role of IL-18 in various autoimmune diseases, such as diabetes mellitus and rheumatoid arthritis [68].

TNF is another important cytokine involved in the activation of local and systemic inflammation, and this transmembrane protein is synthesized by monocytes, macrophages and other cell types, such as lymphocytes and polymorphonuclear leukocytes. TNF is involved in different cellular processes, such as apoptosis, cellular proliferation, and inflammation. TNF induces the synthesis of different inflammatory cytokines, for ex-ample, IL-8, and other chemotactic cytokines, such as monocyte chemoattractant protein-1 (MCP-1), which is involved in the increment of monocytes at the site of inflammation.

TNF and IL-1 are involved in the acute phase of inflammation in combination with IL-6, an important marker of immune system activation, playing a role in thermoregulation, bone homeostasis, and nervous system functionality.

IL-6, secreted from leukocytes activated by an antigenic stimulus, induces B lymphocyte differentiation and specifically modulates growth/arrest mechanisms of different cellular types. The main role of IL-6 is pro-inflammatory but, being a multifunctional cytokine, it could also have anti-inflammatory effects.

In the last 15–20 years, the role played by the different cytokines in the pathogenesis of diseases has been identified, and this knowledge has acquired a strategic relevance for the assessment of effective pharmacological treatments. Indeed, cytokine production and their receptor antagonists represented the targets of a new category of biological drugs that possessed a high specificity compared to previous pharmacological treatments. In the past, for many of these autoinflammatory diseases, there was no specific etiological treatment; most of them were defined as orphan diseases, and the only effective therapy was cortisone. This drug, with anti-inflammatory and immunosuppressive functions, also has important collateral effects, especially in pediatric age since it blocks growth, delays bone development, and causes weight gain and mood instability. Therefore, the identification of cytokines as therapeutic targets has great relevance: examples of this are canakinumab and anakinra, inhibitors of soluble IL-1β and its receptor, respectively; infliximab and certolizumab, which bind TNF-α; tocilizumab, which blocks IL-6 (Table 1) [69,70,71,72,73,74,75,76,77,78,79,80,81,82,83,84,85,86,87,88,89,90,91,92,93,94,95,96,97,98,99,100,101,102,103,104,105,106,107,108,109,110,111,112,113,114,115,116,117,118,119,120,121,122,123,124,125,126].

**Figure 1:**
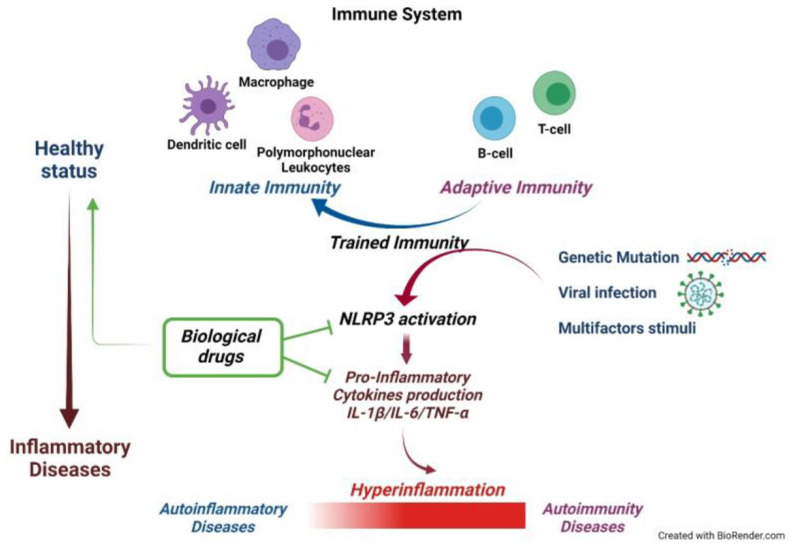
Schematic representation of the link between autoinflammatory and autoimmunity diseases.

This therapeutic approach has promoted the research of specific cytokine markers characteristic of the different autoinflammatory pathologies [127,128]: these studies have also enabled the definition of the cross-talk between the different cytokines and the kinetics of secretion during the activation of inflammation (Figure 1).

## 3. Hyperinflammation and “Cytokine Storm”, Uncontrolled Immune Responses

Hyperinflammatory syndrome seems to have a twofold cause represented by an excessive secretion of pro-inflammatory cytokines and, at the same time, also by a deregulation of the immune response. Uncontrolled hyperinflammation can be caused by viral activation of immunity at the multiorgan level, which, in practice, triggers a real cytokine storm that is not able to effectively allow the activation of the adaptive response, as it has been well evident in SARS-CoV-2 infection [129,130]. In fact, although the spectrum of organs affected by the viral action of COVID-19 is very broad, the negative effects are attributable to the uncontrolled immune response rather than to direct tissue damage caused by the viral infection.

The documented use of the term “cytokine storm’’ dates back to 1993, the year in which Ferrara JL et al. described the response of a transplant against the host [131]. Starting from that evidence, in the following years, this definition was consolidated in the field of transplants [132,133,134], and, subsequently, it was extended to include an uncontrolled inflammatory response in relation to other infectious diseases, with a prevalence in viral infections H5N1 and SARS [135,136,137]. In fact, as for many other viruses, especially SARS, MERS, and H5N1, a cytokine storm is used as a warning signal that indicates a progression of the disease to the clinician. The absence of treatment for a cytokine storm caused by COVID-19 produces immunopathogenic damage that can extend rapidly to organ damage and lead to a fatal outcome [138]. Similarly to all viruses, COVID-19, in order to exert its action, interferes with the innate immune system to promote pathogenicity and alter the immune response of the host. Experimental evidence has shown that in the case of COVID-19, its replication and virulence are related to viroporin proteins E and Orf3a [139,140,141]. Clinical data associated viroporin protein activity with lung damage and an inflammatory state found in patients with SARS-CoV-2 and, in particular, with the secretion of IL-1β. This clinical evidence is experimentally supported by a study conducted on cell and animal models, where viroporin protein deletion was induced, resulting in the reduced secretion of cytokine IL-1β [142].

It has been proven that this viporin protein, indeed, allows the passage through the cell membrane of calcium ions that promote the activation of NLRP3 inflammasomes re-sulting in the production of IL-1β [143,144,145,146]. Therefore, the ionic movements begin the cytokine biosynthesis and potentially can support cytokine storms. In addition, other experimental evidence has shown that the COVID-19 protein Orf3a triggers and activates inflammasome through the efflux of potassium ions and the protein NEK7 or activates NF-kB [147,148,149,150].

Other experimental data have shown that viroporin E can also activate NLRP3 independently from the ion channels through ubiquitination [151].

Finally, as part of the NLRP3 activation in SARS-CoV-2 patients, recent studies support the hypothesis that the P2X7R/NLRP3 component may be involved in immune dysfunction caused by COVID-19 infection [152,153].

This evidence, collected to date on the inflammatory pathogenesis caused by viral infection, shows how the immune system, normally essential to defend us from pathogenic agents, can, however, be a powerful weapon that sometimes damages healthy cells.

During a cytokine storm, the immune system goes out of control, and the final effects risk being more dangerous than the infection itself and may cause tissue damage, organ failure, and eventually death [154].

A cytokine storm is an epiphenomenon associated with several diseases, including malaria [155], lupus, and certain types of arthritis [156], and knowledge of the underlying mechanisms of this state of hyperinflammation has been crucial to try to identify a potential pharmacological strategy against the consequences of COVID-19 infection [157,158,159].

An example is tocilizumab, an antagonist of IL-6, which limits the effects of hyperinflammation and has been described by several studies [160,161,162], likewise anakinra, an inhibitor of the IL-1β receptor [163,164,165].

Of note, the selective inhibition of specific cytokines, during viral infections or other diseases characterized by hyperinflammation, is, however, always evaluated with great caution, as it also inhibits physiological responses of the immune system. In fact, too strict inhibition of several cytokines in some cases could produce the opposite effect, as it could cause the organism not to respond to pathogens [166,167].

## 4. A New Immunity Component: Trained Immunity. What Is Its Role in Autoinflammatory Diseases and Cytokine Storms?

Immune memory has always been closely associated with adaptive immunity, but, in recent years, studies have shown that cells belonging to innate immunity, after an adequate trigger, can effectively contribute to its formation. This immunity component is called trained immunity [168,169,170].

This new part of the immunity system is defined as a reprogramming of the metabolism of innate immunity cells as a response to both endogenous and exogenous stimuli. In particular the cells involved are monocytes and macrophages present in the tissues [171,172] or hematopoietic stem cells at the bone marrow level [173].

At present, the administration of beta glucan [174] and the Bacillus Calmette–Guérin (BCG) vaccine, known as the tuberculosis vaccine, represent the main stimuli able to induce the trained immunity. In particular, the BCG vaccine is considered to be crucial for defense against secondary infections [175,176,177]. Clinical studies have suggested that the BCG is able to induce immunological changes both at the level of innate and adaptive immunity: in particular in innate immunity cells, it induces histone modifications and epigenetic reprogramming at the level of the promoter sites of the genes coding for IL-1, IL-6, and TNF-α [178]. The vaccines and mild infections, indeed, induce the reprogramming of innate immune cells for a consequent strong overall immunologic response to pathogens. The changes induced by the *stimuli*, in turn, activate monocytes and macrophages, which induce the production of cytokines and alter the cellular metabolic state from oxidative phosphorylation to aerobic glycolysis. This condition represents an advantageous situation in dealing with serious infectious diseases, such as COVID-19, because it decreases the rate of morbidity [179]. However, under certain conditions, trained immunity may also have harmful effects. Hyperactivation of the innate immune system for a prolonged period as well as an intense immune reaction to secondary stimuli can represent a high risk of developing chronic inflammatory diseases, such as atherosclerosis. In addition, the phenotype associated with trained immunity was observed in patients with hypercholesterolemia [180].

In some experimental studies on disease models in vivo, the chronicity of the inflammatory state, proper also of trained immunity, has been suggested to be associated with the progression of neurodegenerative diseases [181]. Studies on Alzheimer’s disease models have revealed that chronic systemic inflammation can induce functional and even epigenetic changes in microglia comparable to the systemic condition. These changes are then correlated with abnormal synthesis of beta-amyloid, resulting in damage to the neuronal network associated with a cognitive decline [182].

Trained immunity also plays an ambivalent role compared to cell death in tumor progression; in fact, the conspicuous activation of the immune system is one of the main criteria for the activation of pathways associated with cell death, but, at the same time, chronic inflammation has been associated with tumor pathogenesis in several experimental studies [183].

Moreover, in the delicate balance of innate immunity in the progression of tumor pathogenesis, pro-inflammatory cytokines, such as IL-6 [184] and TNF-α [185,186], play an important role and are secreted by cells that support this part of immunity. These cytokines are identified as markers of carcinogenicity and metastasis in different tumors, such as lung cancer, breast cancer, and oral spinocellular carcinoma.

## 5. Conclusions

The incorrect activation of innate immunity gives rise to heterogeneous forms of auto-inflammatory diseases presenting clinical manifestation due to genetic dysregulation and responses to external stimuli or adaptation to them. Furthermore, recent studies have demonstrated that external insults are able to modulate the innate response, generating a state of hyperinflammation [187].

In fact, it has been noted that vaccines (such as the BCG vaccine) play an important role in training innate immunity, and this evidence could partly help to understand the reason why children, subject to vaccinations in the early years of life, are the category least affected by coronavirus infection [188,189,190].

Moreover, the induction of trained immunity and its effects in the pathogenesis of autoinflammatory diseases has been an important tool in the management of serious viral infections, such as COVID-19 [191]. Based on this evidence, other researchers have also evaluated the role of influenza vaccination as an indirect system useful to increase the body’s defenses against COVID-19 [192,193].

Experimental and clinical evidence on autoinflammatory diseases, even those that are rare, has proved to be extremely useful in addressing the health emergency of the pandemic caused by COVID-19, even simply in preventing/limiting the cascade effects of hyper inflammation and cytokine storms [194,195,196,197,198].

To support these conclusions, indeed, it is notable that AIFA (Italian Medicines Agency) has recently authorized anakinra, an anti-IL-1β drug (already used for rheumatoid arthritis) for the treatment of patients affected by COVID-19, and other studies confirmed the efficacy of biologic drugs for the early comparison to COVID-19 clinical manifestation [199,200,201].

In conclusion, further studies are needed to continue to deepen and explore the pathogenesis of pathologies that derive from innate immunity defects, as they are rich in relevant transversal diagnostic and therapeutic implications and are important for the understanding of the mechanisms of inflammation induced by COVID-19.

## Figures and Tables

**Table 1:** Therapeutic targets of biological drugs.

Cytokine Target	Principal Cell Source Cytokine	Principal Cellular Targets and Biologic Effects	Biological Drug		Structure	Disease
TNF-α	Macrophages, T cells	Endothelial cells. Neutrophils and inflammation activation. Induces apoptosis in many cell types.	TNF inhibitors [69]	ADALIMUMAB [70]	Human Monoclonal Antibody	- plaque psoriasis [71] - psoriatic arthritis [72] - rheumatoid arthritis [73] - hidradenitis suppurativa [74] - non-infectious uveitis [75] - polyarticular juvenile idiopathic arthritis [76] - moderate-to-severe Crohn’s disease and ulcerative colitis [77]
				GOLIMUMAB [78]	Human Monoclonal Antibody	-moderate to severe ulcerative colitis [79] - rheumatoid arthritis, psoriatic arthritis/spondyloarthritis [80]
				ETANERCEPT [81]	TNF- α receptor-Fc fusion	- plaque psoriasis [82] - psoriasis [83] - juvenile idiopathic arthritis [84] - psoriatic arthritis [85] - rheumatoid arthritis [86]
				INFLIXIMAB [87]	Chimeric monoclonal antibody	- idiopathic inflammatory myopathies [88] - moderate-to-severe Crohn’s disease and ulcerative colitis [77] - Behçet’s disease [89] - rheumatoid arthritis [90] - ankylosing spondylitis [91] - psoriasis [92]
				CERTOLIZUMAB-pegol [93]	Mouse Monoclonal Antibody (Fab’ fragment)	- Crohn’s Disease [94] - rheumatoid arthritis [95] - psoriasis [96]
IL-1 β	Macrophages, endothelial cells, epithelial cells	Endothelial cells, hypothalamus, liver	IL-1 β inhibitors [97]	ANAKINRA [98]	Interleukin-1 receptor antagonist	- Still’s disease [99] - rheumatoid arthritis [100] - cryopyrin-associated periodic syndrome [101] - autoinflammatory disorders [102,103]
				CANAKINUMAB [104]	Human Monoclonal Antibody	-autoinflammatory disease [105] - hyperimmunoglobulin D syndrome [106] - Familial Mediterranean Fever [107] - juvenile idiopathic arthritis [108]
				RILONACEPT [109]	Interleukin-1 receptor antagonist	- chronic inflammatory disorders [110] - cryopyrin-associated periodic syndromes [111] - recurrent pericarditis [112] - gout [113]
IL-6	Macrophages, endothelial cells, T cells	Liver, B cells	IL-6 inhibitors [114]	TOCILIZUMAB [115]	IL-6 receptor monoclonal antibodies	- rheumatoid arthritis [116] - giant cell arteritis [117] - Still’s disease [118] - juvenile idiopathic arthritis [119] - Macrophage activation syndrome [120]
				SARILUMAB [121]	IL-6 receptor monoclonal antibodies	- moderate-to-severe Rheumatoid Arthritis [122]
				SILTUXIMAB [123]	IL-6 monoclonal antibodies	- Castleman’s disease [124] - non-Hodgkin lymphoma [125] - autoimmune diseases [126]

## Data Availability

Not applicable.
